# Protection Effect of Zhen-Wu-Tang on Adriamycin-Induced Nephrotic Syndrome via Inhibiting Oxidative Lesions and Inflammation Damage

**DOI:** 10.1155/2014/131604

**Published:** 2014-04-09

**Authors:** Chun-ling Liang, Jun-biao Wu, Jie-mei Lai, Shu-fang Ye, Jin Lin, Hui Ouyang, Janis Ya-xian Zhan, Jiu-yao Zhou

**Affiliations:** School of Chinese Materia Medica, Guangzhou University of Chinese Medicine, Guangzhou, Guangdong 510006, China

## Abstract

Zhen-wu-tang (ZWT), a well-known formula in China, is widely used to treat chronic kidney diseases. However, very little information on ZWT's mechanism of action is currently available. In this study, we investigated the possible protective role and underlying mechanism of ZWT on nephrotic syndrome (NS) induced by Adriamycin (intravenous injection, 6.0 mg/kg) in rats using biochemical and histopathological approaches. ZWT decreased urine protein excretion and the serum levels of total cholesterol, triglycerides, blood urea nitrogen, and creatinine significantly in diseased rats. A decrease in plasma levels of total protein and albumin was also recorded in nephropathic rats. Pathological results show an improved pathological state and recovering glomerular structure in ZWT treatment groups. ZWT decreased renal IL-8 level but increased renal IL-4 level. In addition, rats subjected to ZWT exhibited less IgG deposition in glomerulus compared with model group. RT-PCR results showed that ZWT decreased the mRNA expression of NF-**κ**B p65 and increased the mRNA expression of I**κ**B. Furthermore, ZWT reduced the level of MDA and increased SOD activity. These results demonstrated that ZWT ameliorated Adriamycin-induced NS in rats possibly by inhibiting Adriamycin-induced inflammation damage, enhancing body's antioxidant capacity, thereby protecting glomerulus from injury.

## 1. Introduction

Nephrotic syndrome (NS) is the triad of proteinuria, low serum albumin, and edema. The disease is seen mostly in boys with predominance in children from the Asian subcontinent [1]. The global pandemic of NS is progressing at an alarming rate and severely diminishes the quality of life for millions [2]. Despite the changing face of NS, treatment options and resources remain woeful. The first line of treatment uses old but important approaches to reduce kidney tissue damage by trying to control the underlying conditions of NS. For example, patients with hypercholesterolemia secondary to NS always treated with lip-lowering agents and with edema secondary to salt and water retention can be controlled with the judicious use of diuretics. Venous complications associated with NS can be treated with anticoagulants [3]. Of all the therapeutics, hormone therapy is the most commonly used, but it has many side effects and is easy to recrudesce. If the treatment fails, the patient begins to experience kidney failure. Traditional Chinese prescription has been commonly recognized as safe and effective in the treatment of chronic kidney disorders in China and Japan with its various effects made by a variety of chemical components multilinked and multitargeted in the body.

NS has been associated with a wide variety of abnormalities in the immune response, especially in cell mediated immunity which supposed that soluble factors secreted by T cells may form the critical upstream signal for initiating glomerular changes in NS [4]. The occurrence of NS, proteinuria, and renal pathologic changes are associated with the change in Th1/Th2 in favor of Th2 lymphocytes [5]. The reiterative active renal inflammation is associated with the development of proteinuria, renal dysfunction, glomerular cell proliferation, and extracellular matrix components expansion and prolonged renal fibrosis [6, 7]. Therefore, inhibiting the activation of renal inflammation could be the therapeutic target of protecting renal lesion in NS. NF-*κ*B, a ubiquitous transcription factor, governs the expression of genes encoding for cytokines, chemokines, growth factors, adhesion molecules, and other factors involved in the immune and inflammatory response. A series of correlations between the activation of NF-*κ*B and onset of both immune- and nonimmune-mediated glomerular injury implicate dysregulation of the NF-*κ*B pathway in its pathogenesis [8–10]. Therefore, we postulated possible involvement of NF-*κ*B in the pathogenesis of NS. Antioxidant therapy is another important strategy for NS treatment and is considered to be an important pathogenic mechanism. Reactive oxygen species (ROS) are continuously produced physiologically and play an important role in the expression of cell functions such as transmission of impulse information. However, excessive production of ROS would act as mediators of adverse events such as inflammation, necrosis, and apoptosis. In the kidney, ROS are important causes of acute and subacute renal failure in most cases. Also, in certain animal models of NS, superoxide anions and hydroxyl radicals are the main causes of nephrotoxicity [11]. It has also been reported that renal failure that results from increased ROS and decreased antioxidant enzyme can be prevented by antioxidant therapy [12, 13].

Zhen-wu-tang (ZWT) is a classic prescription to treat chronic kidney disease (CKD) with the paths of Wen* yang* and Li Water, which is recorded in* Treatise on Febrile Diseases* [14]. As we know, edema is the most common presenting feature in CKD. It becomes detectable when the fluid retention exceeds 3% of the weight of the body [15]. ZWT has been used as a remedy for various kidney diseases with the efficacy to relieve relative symptoms manifested by edema, dysuria, and oliguria in clinics in China and Japan [16]. Furthermore, the decrease in levels of collagen IV, fibronectin, and laminin in the extracellular matrix of the fetal glomerular mesangial cells has been observed after treatment by ZWT [17]. However, the effect of ZWT on Adriamycin- (ADR-) induced NS has not been well explored. In this study, we will investigate the immune regulation and antioxidation effects of ZWT on rats with NS induced by ADR. The aims of the present study are to investigate the protective effects of ZWT on ADR-induced NS and to discuss possible underlying mechanism of action in rat model.

## 2. Methods and Materials

### 2.1. Drugs and Drug Treatment

#### 2.1.1. Plants Material and Drugs

ZWT is composed of five herbal medicines: Aconiti Lateralis Radix Praeparata (the lateral radix of* Aconitum carmichaelii* Debx.), Zingiberis Rhizoma Recens (rhizome of* Zingiber officinale* Rosc.), Atractylodis Macrocephalae Rhizoma (radix of* Atractylodes macrocephala* Koidz.), Paeoniae Radix Alba (radix of* Paeonia lactiflora* Pall.), and Poria (sclerotium of* Poria cocos *(Schw.) Wolf.). The dried raw materials of the herbs were purchased from the Zhixin Medicinal Materials Company, Guangzhou, China (Lot. YPA110001, 120301, 120301, and 120401, resp.). All herbal medicines were authenticated by Professor Qiu-Zhen Zhang at Guangdong Chinese Medicine Museum. To assure the quality control, the materials were validated according to the Chinese Pharmacopeia. Dexamethasone was purchased from the Second Affiliated Hospital, Guangzhou University of Chinese Medicine, Guangzhou, China (Lot. 111001).

#### 2.1.2. Preparation of ZWT

Amounts of Aconiti Lateralis Radix Praeparata, Poria, Atractylodis Macrocephalae Rhizoma, Paeoniae Radix Alba, and Zingiberis Rhizoma Recens were mixed according to a ratio of 3 : 3 : 2 : 3 : 3. All materials were soaked with eight times distilled water for one hour and then boiled with distilled water for 2 h. For second extraction, the residue from the first extraction was filtered, and the same extracting condition was applied. After being repeated for three times, the mixture of the filtrates was concentrated to the concentration of 2.4 g raw materials per milliliter. The extract was kept at 4°C and dissolved in distilled water before use.

### 2.2. Chemicals and Reagents

ADR was purchased from Shenzhen Main Luck Pharmaceuticals Inc. (Lot. 0901E); Urinalysis Reagent Strip from Acon Biotech (Hangzhou) Co., Ltd. (Lot. 201109144); Coomassie (Bradford) Protein Assay Kits from Nanjing Jiancheng Bioengineering Institute (Lot. 20120413 and 20120604); Interleukin-4 and Interleukin-8 ELISA kits for rats from Abcam (HK) Ltd. (Lot. ISC10330EIA-2121R and -2135-R, resp.), Rabbit Anti-Rat IgG/FITC from Beijing Biosynthesis Biotechnology Co., Ltd. (Lot. 990603), SOD and MDA Assay Kits from Nanjing Jiancheng Bioengineering Institute (Lot. 20120523 and 20120324); Trizol (9108, Lot. number AK8106), RT Reagent Kit (RR047A, Lot. AK2003), and SYBR Premix ExTaq II (RR820A, Lot. number AK4401) were from Takara, Dalian, China; the primers for NF-*κ*B p65, I*κ*B, and GAPDH were synthesized by Sangon Biotech (Shanghai) Co., Ltd.

### 2.3. Animals and Treatments

Fifty adult male special pathogen free Sprague Dawley rats (Guangzhou University of Chinese Medicine Research Center for Experimental Animal), which weighed (200 ± 20) g, with urine protein qualitative tests showing negative results (Urinalysis Reagent Strip) (Certificate number SCXK (Guangdong) 2008-0020), were used in the experiments. The rats were housed in an air-conditioned room at 25± 2°C and 65% humidity, with a 12 h light/12 h dark cycle (Certificate number 2008-0085). During the experiments, all animals were given* ad libitum* to standard laboratory rats chow and water. After 1 week of acclimatization, the rats were divided into five groups (ten animals in each group): control group; model group; ZWT groups (ADR + ZWT 24.0 g/kg and ADR + ZWT 12.0 g/kg); DXM group (ADR + DXM); the dosages of ZWT were chosen referring to the clinical dosage and the result of our preliminary experiment. The rats were intravenously injected with ADR (6.0 mg/kg, dissolved in C). The normal group was injected with saline (1.0 mL/100 g) only. One week after injection, the rats were administrated distilled water (control group and model group) or ZWT by an oral gavage method once daily for 28 days. All experiments conformed to the European Community Guidelines and the regulations of the National Institute of Health of US.

### 2.4. Measurement of Uric Protein

To measure urine protein levels, 24 h urine samples were collected using metabolic cages on days 0, 7, 14, 21, 28, and 35. All rats were forbidden from food and free access to water during the course of sample collection. Urinary protein was determined by colorimetric method.

### 2.5. Blood Sampling and Tissue Removal

On day 35, all animals were sacrificed for blood sample and renal tissue. The blood samples were obtained from abdominal aorta one hour after gavage administration under chloral hydrate anesthesia. Serum was separated by centrifugation at 4°C at 3500 rpm for 15 min and stored at −20°C for biochemical analysis. After exsanguination, the kidneys were rapidly removed and weighted. The two kidneys were individually divided into two parts. The upper pole of the left kidney was treated for light microscopy; the lower pole of the left kidney was divided into four parts, quickly frozen with liquid nitrogen, and kept in −80°C for the assay of IL-4 and IL-8. The upper pole of the right kidney was frozen with liquid nitrogen and then kept in −80°C for immunofluorescence assay of IgG. The lower pole of the left kidney was divided into four parts; one was for electron microscopy, and the rest were quickly frozen with liquid nitrogen and kept in −80°C for gene analysis.

### 2.6. Measurement of Blood Biochemical Parameters and IL-4 and IL-8 in Renal Tissue

Serum cholesterol (CHOL), triglycerides (TRIG), serum albumin (ALB), serum total protein (TP), creatinine (Scr), and blood urea nitrogen (BUN) levels were analyzed using automatic clinical chemistry analyzer. Plasma SOD and MDA levels were tested according to instructions of assay kits. Renal tissue was homogenized in saline (100 mg tissue/mL) with homogenizer and then centrifuged at 4°C at 3000 g for 20 min. The supernatant was kept at −20°C for the assay of interleukin-4 and interleukin-8 levels using ELISA kits.

### 2.7. Determination of Renal IgG

Renal cortical tissues were cut into 5 *μ*m sections using freezing microtome and then operated as the following steps: they were fixed in acetone for 10 min, washed in phosphate buffer saline for 3 min, stained with 1 : 50 diluted rabbit anti-rat IgG antibody (FITC labelled) at room temperature for 30 min, washed in phosphate buffer saline twice, 5 min each time, washed in distilled water for 1 min, and wet mounted with 50% glycerin. These finished sections were examined under inverted fluorescence microscopy and eight micrographs were obtained at random with magnification of 400x. The fluorescence signal was quantified using image processing software (Image J 1.47), with eight micrographs for each section and six sections for each group. The result was expressed as average density (/pixel) (average density = integrated density/area).

### 2.8. Light Microscopy

The cortical tissues were fixed with 10% neutral formalin phosphate buffer, dehydrated through a graded alcohol series, and embedded in paraffin, and then they were cut into 5 *μ*m sections and stained with hematoxylin and eosin (H&E) and examined under the light microscope (TE2000, Nikon, Japan).

### 2.9. Electron Microscopy

A portion of cortical tissues was cut into 1 mm cubes, fixed in 2.5% glutaraldehyde, and postfixed in 1% osmium tetroxide. The samples were dehydrated through a graded alcohol series and embedded in Epon 812. Four ultrathin sections (60 nm) were cut with a diamond knife continuously for each sample and stained with uranyl acetate and lead citrate. The sections were examined under the electron microscope (JEM100CX-a, Japan) at 60 kv, ×5000 magnification.

### 2.10. NF-*κ*B p65 and I*κ*B Genes mRNA Determination

The total RNAs from different experimental groups were obtained by Trizol method. The concentration of RNA was determined by an absorbance at 260 nm and the purity of the RNA was evaluated by measuring the A260/A280 ratio. RNA was reverse transcribed to cDNA using the Takara reverse transcription reagent with gDNA Eraser. All RNA samples performed gDNA removal step (42°C, 30 min). Reverse transcription was performed at 37°C 15 min and 85°C 5 s. PCR was performed with the SYBR Green PCR Master Mix using the following oligonucleotide primers: NF-*κ*B (5′-ACGATCTGTTTCCCCTCATCT-3′, antisense 5′-TGGGTGCGTCTTAGTGGTATC-3′); I*κ*B (5′-GAGGAAATACCCCTCTCCATCT-3^′,^ antisense 5′-GCCCTGGTAGGTTACTCTGTT-3′); GAPDH (5′-ACAGCAACAGGGTGGTGGAC-3′, antisense 5′-TTTGAGGGTGCAGCGAACTT-3′). The real-time quantitative PCR used ABI7500 (Applied Biosystems, USA) and the cycling program was set at 1 cycle of predenaturation at 95°C for 30 s, and then 40 cycles at 95°C for 10 s, 60°C for 34 s, and then melting curve was analyzed. All the RT-PCR experiments were conducted strictly according to the rules of the MIQE.

### 2.11. Statistical and Analysis

Datas are presented as mean ± SD. Statistical analysis was performed with the independent-samples *t*-test by SPSS 17.0 software. Differences were considered significant when *P* < 0.05.

## 3. Results

### 3.1. General Conditions

On the third day of tail intravenous injection of ADR, proteinuria in the rats was increased gradually. Rats in model group presented lower food intake and less activity compared with those in control group. However, rats in treatment groups showed a better mental condition, raised movement and food intake, and a better hair sheen.

### 3.2. Urinary Protein Analysis

**Figure 1:**
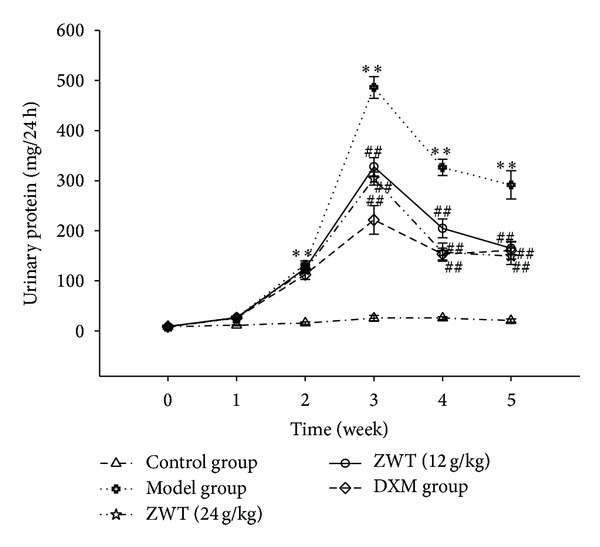
ZWT decreased the increased 24 h urinary protein induced by ADR. Data were expressed as mean ± SD, *n* = 8. Vertical bars represent standard errors of the means, and asterisks and pound signs designate significant differences: ^#^ *P* < 0.05 and ^##^ *P* < 0.01 versus model and *P < 0.05 and **P < 0.01 versus control.

Proteinuria is one of the important characteristics in ADR-induced rat nephrotic syndrome. In our study, we demonstrated that a single injection of ADR at 6.0 mg/kg increased 24 h uric protein excretion three days after the injection and the proteinuria reached a maximum of three weeks after the injection. Interestingly, treating rats with ZWT at the dosages of 24.0 g/kg/d and 12 g/kg/d for 3 weeks resulted in significant declines in the levels of 24 h uric protein in parallel to the model group (*P* < 0.01) (Figure 1).

### 3.3. Blood Biochemical Parameters

As depicted in Table 1, compared with control group, the model group displayed increased serum CHOL and TRIG, Scr, and BUN (*P* < 0.01) but decreased levels of serum TP and ALB (*P* < 0.05). These phenotypes were similar to the clinical symptoms of nephrotic syndrome. Furthermore, ZWT at the dosages of 24.0 g/kg/d and 12.0 g/kg/d for 28 days significantly prevented the increase in serum CHOL, TRIG, Scr, and BUN and the decrease in serum TP and ALB.

### 3.4. Changes in the Kidney Shape

**Figure 2:**
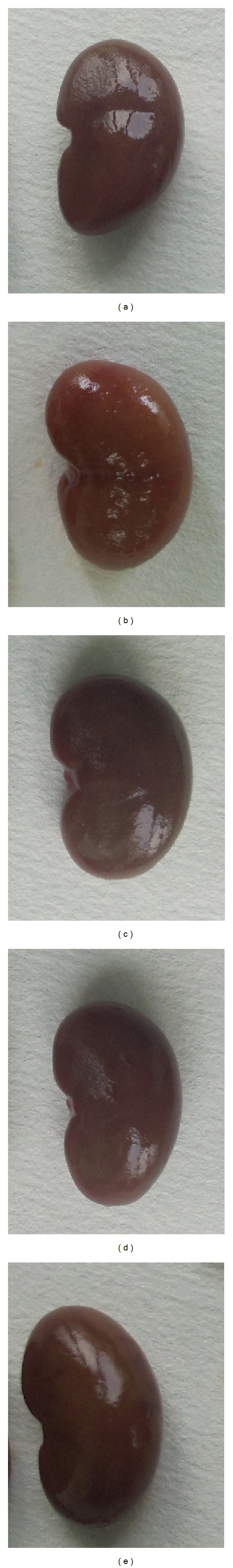
Macroscopic morphology of the kidneys among five groups: (a) control group (treated with saline); (b) model group (treated with ADR); (c) ZWT group (treated with ZWT, 24.0 g/kg); (d) ZWT group (treated with ZWT, 12.0 g/kg); (e) DXM group (treated with DXM, 0.9 mg/kg).

Long-term NS may induce changes in the conditions of kidney. In the present study, kidney of ADR-treated rats showed severe edema and less luster compared with the control group. The kidney in ZWT treated group (24.0 g/kg/d and 12.0 g/kg/d) presented more dense shape and redder look (Figure 2). Moreover, as shown in Table 2, the ration of absolute kidney weight (KW) to BW was increased in ADR model rats. Treatment with ZWT prevented the increase of KW/BW.

### 3.5. IL-4 and IL-8 in Renal Tissue and SOD and MDA in Plasma

**Figure 3:**
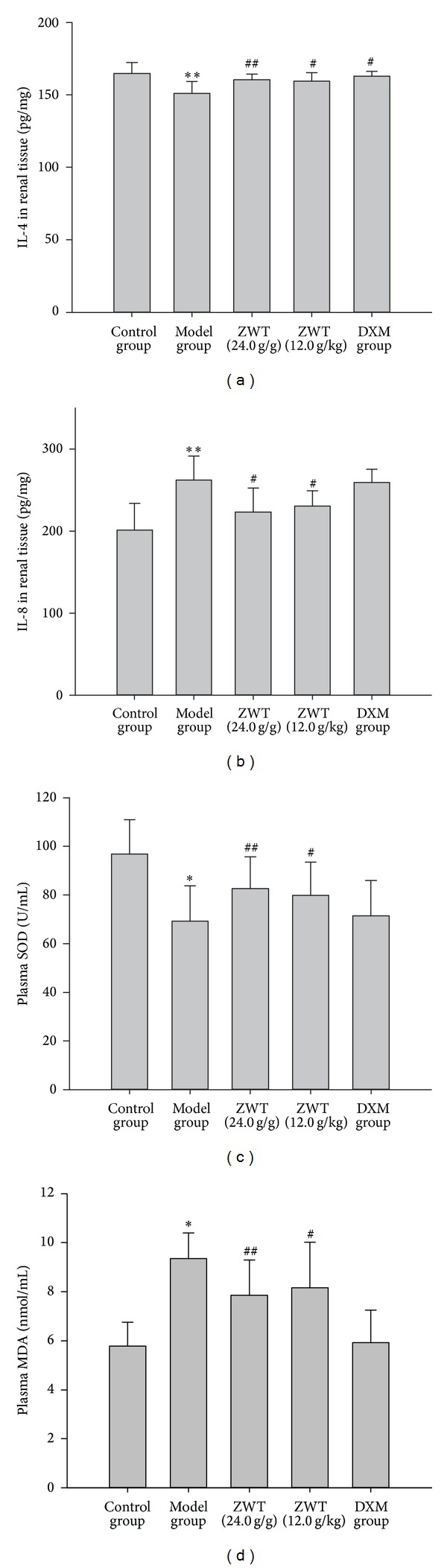
The effects of ZWT (g/kg) on IL-4 and IL-8 in renal tissue and antioxidant index in plasma. (a) IL-4 in renal tissue; (b) IL-8 in renal tissue; (c) plasma SOD; (d) plasma MDA. DMX: dexamethasone (0.9 mg/kg); data were expressed as mean ± SD, *n* = 8. Vertical bars represent standard errors of the means. Asterisks and pound signs designate significant differences: ^#^ *P* < 0.05 and ^##^ *P* < 0.01 versus model and *P < 0.05 and **P < 0.01 versus control.

In our study, we found that treatment with ZWT at the dosages of 24.0 g/kg/d and 12.0 g/kg/d for 28 days could significantly prevent the increase of renal IL-8 and the decrease of renal IL-4 induced by ADR (Figures 3(a) and 3(b)). ZWT could boost antioxidant power and decrease free radicals damage in NS rat, raise the plasma SOD activity, and lower the content of plasma MDA (Figures 3(c) and 3(d)).

### 3.6. Pathologic Changes of Kidney

#### 3.6.1. Determination of Renal IgG

**Figure 4:**
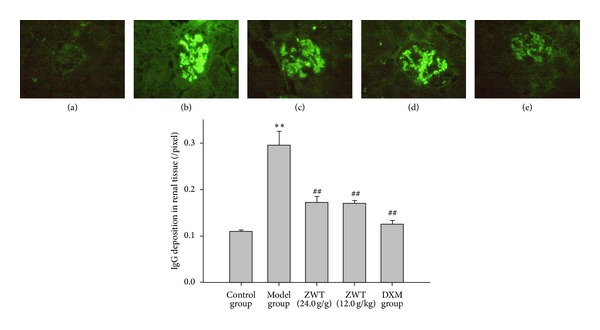
ZWT decrease the IgG deposition in renal tissue. Magnification, ×400. (a) Control group (treated with saline); (b) model group (treated with ADR); (c) ZWT group (treated with ZWT, 24.0 mg/kg); (d) ZWT group (treated with ZWT, 12.0 mg/kg); (e) DXM group (treated with dexamethasone, 0.9 mg/kg). Data were expressed as mean ± SD, *n* = 6. Vertical bars represent standard errors of the means. Asterisks and pound signs designate significant differences: ^#^ *P* < 0.05 and ^##^ *P* < 0.01 versus model and *P < 0.05 and **P < 0.01 versus control.

Using immunofluorescent assay, we found that the control group showed extremely weak fluorescence, but ADR-treated rats showed strong fluorescence at low magnification (*P* < 0.01). Further, treatment with ZWT decreased the strong fluorescence induced by ADR (*P* < 0.01) (Figure 4).

#### 3.6.2. Light Microscopy and Electron Microscopy

**Figure 5:**
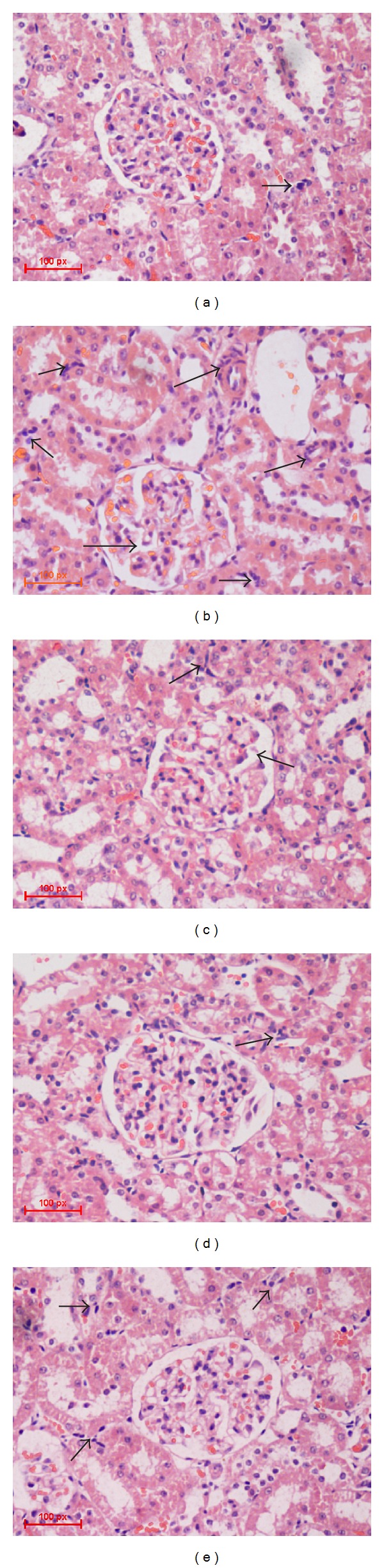
Morphology change in glomerulus under light microscopy. Transmission light microscope; magnification, 10 × 40. (a) Control group (treated with saline); (b) model group (treated with ADR); (c) ZWT group (treated with ZWT, 24.0 g/kg); (d) ZWT group (treated with ZWT, 12.0 g/kg); (e) DXM group (treated with dexamethasone, 0.9 mg/kg).

**Figure 6:**
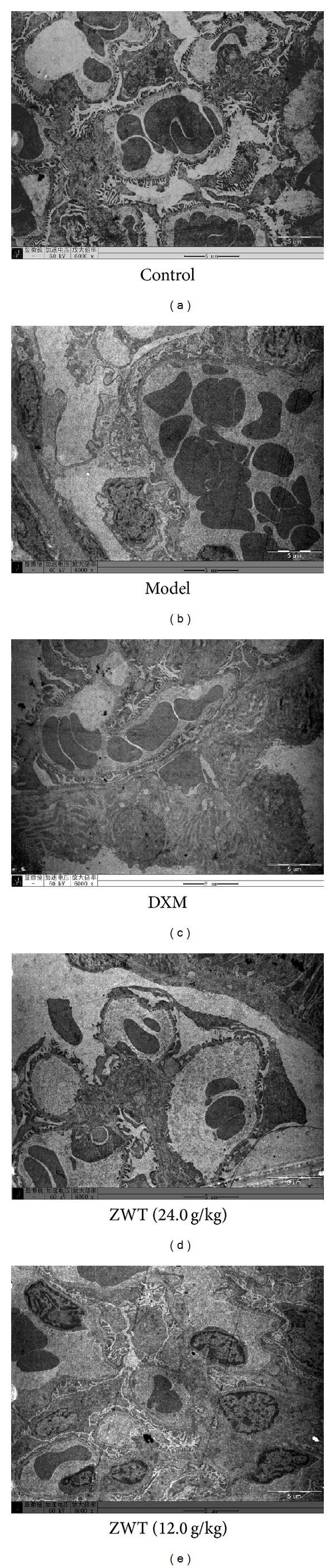
Morphology change in glomerulus under light microscopy. Transmission electron microscope; magnification, ×6000. (a) Control group (treated with saline); (b) model group (treated with ADR); (c) ZWT group (treated with ZWT, 24.0 g/kg); (d) ZWT group (treated with ZWT, 12.0 g/kg); (e) DXM group (treated with dexamethasone, 0.9 mg/kg).

Renal pathological examination is a fast, clear, and direct method in diagnosis of nephrotic syndrome. In model group, glomerular extracellular matrix (ECM) accumulation and glomerular mesangial cell (GMC) proliferation and base-membrane thickness were observed. Further, foot processes were wide effacement and the width of foot process was much larger than that of normal control rats (Figure 5). Under the light microscopy, renal tubule in model group was dropsical and Bowman's space was larger than that in control group. And there were less cells in glomerulus in model group than those of control group. At high magnification, the lymphocytes and neutrophils cells are seen around a renal tubule. Treatment of NS rats with ZWT reduced the edema and inflammatory cell infiltration, ameliorated the effacement of foot processes, reduced the lysosomes deposition in foot process, and reduced the edema of glomerulus (Figure 6).

### 3.7. Effects of ZWT on NF-*κ*B p65 and I*κ*B mRNA Expressions

NF-*κ*B plays a key role in the regulation of cytokine expressions. Because most cytokines levels in NS are partly or predominantly regulated by NF-*κ*B, we postulated possible involvement of NF-*κ*B in NS. In the study, the results showed that the mRNA expression of NF-*κ*B p65 in the renal tissue of ADR-induced NS rats was highly upregulated and that of I*κ*B was highly downregulated compared with the control group. However, ZWT could decrease the expression of renal NF-*κ*B p65 but increase the expression of I*κ*B (*P* < 0.01) (Table 3).

## 4. Discussion

ZWT is a blended traditional Chinese medicine specifically for the treatment of various renal diseases. The present study demonstrated that ZWT improved ADR-induced nephrotic syndrome. NS is a series of clinical symptoms, including proteinuria, hypoalbuminemia, edema, and hyperlipidemia [18]. Proteinuria is a hallmark risk factor for that causes most of the subsequent symptoms of NS [19]. Podocyte, model group, is a kind of highly differentiated cells, forming multiple interdigitating foot processes. It is interconnected by the slit diaphragms and covers the glomerular basement membrane surface [20]. It is recognized that the dysfunction of glomerular podocyte and the subsequently cellular death act as the driving forces behind disease initiation and progression, respectively [21]. Extensive effacement of podocyte foot processes is the key morphologic change noted in NS and podocyte damage can result in serious proteinuria. Therefore, it is a central target to inhibit the podocyte from being injured to maintain renal function [22]. In the present study, the foot processes of podocyte were severely effaced in ADR-treated rats at week 5, and proteinuria appeared three days after injection of ADR; treatment with ZWT significantly ameliorated the effacement of foot processes and reduced proteinuria. These results suggested that ZWT improved the podocyte injury in ADR-induced nephrotic syndrome.

The renal function is closely associated with urine protein excretion level and a direct reflection of disease's progression [23]. The concentrations of Scr and BUN, which are two important indexes to reflect renal function, depend on the glomerular filtration rate (GFR). Renal dysfunction reduces the capability of filtering them, and their levels then rise [24]. In this study, the rats did show a notable increase in the Scr and BUN after 5 weeks following injection of ADR, indicating that renal filtrating function was being destroyed by ADR. But the results also showed that ZWT could decrease the levels of BUN and Scr in serum, indicating that ZWT could enhance renal function by reducing the synthesis or increasing the excretion of BUN and Scr in ADR-induced NS rats.

Dyslipidemia is also one of the main phenotypes in ADR-induced NS in the rats. Consistent with previous reports [25], our present study confirmed that ADR increased plasma levels of TRIG and CHOL in rats. The mechanism involved in the pathogenesis of dyslipidemia is still unclear. Hutchison reported that abnormal glomerular permeability to plasma proteins and high density lipoprotein increased biosynthesis of CHOL and TRIG in liver and lipid metabolism disorders, but reduced serum oncotic pressure contributes to hyperlipidemia [26]. In the present study, treatment with ZWT also improved ADR-induced hypertriglyceridemia and hypercholesterolemia. Furthermore, ZWT increased the level of serum TP and ALB improving the hypoproteinemia induced by ADR. All the data demonstrated that ZWT possesses a protective effect on ADR-induced NS by preventing proteinuria, protecting renal function, and ameliorating foot processes effacement. After that, we investigated immune regulation and antioxidation effects of ZWT on ADR-induced NS in rats to reveal its deep therapy mechanism on NS.

A possible role for Th2 cytokines in the induction of proteinuria in minimal change nephropathy (MCN) is supported by clinical observations, such as the association of MCN with atopy and the apparent induction of MCN by allergic events in some patients [27, 28]. IL-4, a pleiotropic cytokine, is produced by appropriately stimulated CD4^+^ Th2 lymphocytes. IL-4 is the primary cytokine for the spontaneous production of IgE and IgG by peripheral blood mononuclear cells, which is associated with immune complex depositing at glomerular basement membrane. However, its role in NS is controversial. In some researches, it was reported that IL-4 was increased in NS rats [29], and IL-4 decreased transepithelial electrical resistance of monolayers of glomerular visceral epithelial cells in rats dose dependently, which suggested that IL-4 could exert specific effects on glomerular visceral epithelial cells function [30]. In some clinical investigations of NS patients, it was found that IL-4 was decreased during relapse and increased in patients with long-term remission [31]. In the present study, the results showed a decreased level of renal IL-4 in ADR-treated group, which was prevented by ZWT. The difference may result from the different stages of disease. For that, IL-4 can also act as anti-inflammatory cytokines. The mechanisms responsible for the anti-inflammatory effects of IL-4 are attributable to the suppression of tumor necrosis factor-*α* (TNF-*α*) production by macrophages and upregulation of anti-inflammatory cytokines such as TGF-*β* [32, 33]. We hypothesized that IL-4 may act as protective effect on NS in later stages. IL-8 is an important chemokine that acts on various inflammatory mediators including neutrophil granulocyte, T-lymphocytes, and basophilic granulocyte. The main biological effect of IL-8 is accelerating chemotaxis of neutrophil granulocytes and IL-8 activity is positively associated with the inflammatory cell infiltration in lesions area [34]. In the present study, the lymphocytes and neutrophils cells are seen around a renal tubule under the light microscopy. In addition, IL-8 level in renal tissue was increased in ADR-treated group compared with control group. That was consistent with previous reports that the serum IL-8 levels of the nephrotic phase were significantly lower than those of remission phase and was positively correlated with proteinuria [35, 36]. Further, patients of NS always have severe immune deposition in glomerular mesangium which is defined as a late-stage symptom. Immune deposition can stimulate the release of local proteases and activate the complement cascade, producing C5-9 attack complex, damaging glomerular structure and producing proteinuria [37]. Immunofluorescence stain showed severe IgG deposition in glomerulus in ADR-treated rats. Renal IgG deposition in ZWT-treated rats was significantly reduced compared with ADR-treated rats. These data suggest that ZWT may ameliorate kidney injury, at least in part, by modulating the balance between inflammatory and anti-inflammatory responses.

NF-*κ*B is a transcription factor activated by cell surface receptor signaling to meet stress and inflammatory responses, regulating key cellular processes such as inflammation, innate and adaptive immunity, and cell growth and survival [38]. Notably, the NF-*κ*B family of transcription factors has been shown to regulate various aspects of T-cell, including Th1, Th2, Th17, Th9, and Tfh cells [39]. It is critical in modulation of the immune response through the transcriptional regulation of cytokine (IL-4, IL-2, IL-6, IL-12, IFN-L-6, IL-CSF, and G-CSF) and chemokine (IL-8 and C3) expression [8, 40]. In addition, NF-*κ*B is sequestered in the cytoplasm bound to I*κ*B; phosphorylation of I*κ*B releases active NF-*κ*B, which translocates to the nucleus to induce an extensive range of target genes [41]. In response to this, we asked whether a direct causal link between NF-*κ*B activation and ADR induced NS and whether ZWT might regulate this pathway. To test this, we examined NF-*κ*B (p65) and I*κ*B mRNA expression in kidneys of a rat NS model induced by ADR. In our experiment, NF-*κ*B (p65) mRNA expression in model group was significantly upregulated, but I*κ*B mRNA expression was significantly downregulated compared with control group. And the results are consistent with what has been reported. The results suggest that the activation of NF-*κ*B pathway was involved in the pathogenesis of NS. ZWT could inhibit the activation of NF-*κ*B and prevent kidneys from being injured.

Antioxidant therapy is another strategy for NS treatment and is considered to be an important pathogenic mechanism. Oxidative stress develops from an imbalance between oxygen free raddical (ORF) production and reduced antioxidant defenses, such as SOD, CAT, glutathione, and glutathione peroxidase [42, 43]. ORF could result in serious DNA damage and lipid peroxidation and thus damage the glomerular filtration barrier and promote renal cell apoptosis and senescence, decreased regenerative ability of cells, and fibrosis [44]. MDA is an important end-product generated by lipid peroxidation and has been used to demonstrate increased oxidative stress during chronic kidney disease [45]. In our study, rats in ADR-treated group presented with high level of plasma MDA which indicated an increased lipid peroxide concentration [46, 47]. ZWT decreased plasma MDA and inhibited the lipid peroxidation. A major mechanism of resistance to ROS is antioxidase enzymes like SOD. Rat treated with ZWT has increased plasma SOD compared with that of model group. We concluded that ZWT could enhance the activity of endogenous antioxidant enzymes to prevent oxygen free radical damage.

## 5. Conclusions 

Taken together, we propose that ZWT could ameliorate the proteinuria, low serum albumin, hypercholesterolemia, and loss of kidney function of NS rats. These data provide direct evidence for ZWT-treated nephrotic syndrome, at least in part, by modulating the balance between inflammatory and anti-inflammatory responses, enhancing antioxidant capacity and the elimination capacity of ROS. However, the active compounds in ZWT responsible for its treatment are not revealed at present, and its more protection mechanisms are also not clear. Therefore, we will continue to investigate further. Network pharmacology and membrane immobilized chromatography would be involved in our follow-up study.

## Figures and Tables

**Table 1:** Improvement of blood biochemical parameters by ZWT (*n* = 8, mean ± SD).

Groups	CHOL (mmol/L)	TRIG (mmol/L)	TP (g/L)	ALB (g/L)	BUN (*μ*mol/L)	Scr (mmol/L)
Control	0.80 ± 0.20	0.53 ± 0.22	293.1 ± 16.9	308.5 ± 25.2	4.49 ± 0.86	26.96 ± 8.00
Model	3.46 ± 0.90^##^	2.91 ± 0.86^##^	268.0 ± 33.0^##^	279.5 ± 33.7^##^	7.50 ± 0.77^##^	47.58 ± 5.20^##^
ZWT (24.0 g/kg)	2.28 ± 0.82**	1.46 ± 0.39**	264.4 ± 20.0**	282.7 ± 82.5**	6.33 ± 0.86*	37.72 ± 5.18*
ZWT (12.0 g/kg)	2.69 ± 0.71**	1.45 ± 0.31**	283.3 ± 19.5**	307.4 ± 20.2**	6.80 ± 0.63**	38.80 ± 7.82*
DXM (0.9 mg/kg)	1.76 ± 0.66**	1.51 ± 0.90*	241.2 ± 17.8**	217.9 ± 14.2**	6.09 ± 1.21*	36.41 ± 8.00**

^#^ *P* < 0.05 and ^##^ *P* < 0.01 versus control; *P < 0.05 and **P < 0.01 versus model; CHOL: serum total cholesterol; TRIG: serum triglycerides; TP: serum total protein; ALB: serum albumin; BUN: blood urea nitrogen; Scr: serum creatinine.

**Table 2:** ZWT decrease the ration of absolute kidney weight to body weight (*n* = 10, mean ± SD).

Groups	KW (g)	BW (g)	KW/BW (10^−3^)
Control	2.36 ± 0.18	352.8 ± 28.6	6.70 ± 0.62
Model	2.46 ± 0.21	297.6 ± 32.2	8.08 ± 0.91^##^
ZWT (24.0 g/kg)	2.31 ± 0.21	327.2 ± 24.2	7.10 ± 0.47**
ZWT (12.0 g/kg)	2.32 ± 0.18	328.4 ± 27.6	7.03 ± 0.32**
DXM (0.9 mg/kg)	1.88 ± 0.18	258.1 ± 21.4	7.03 ± 0.32**

^#^ *P* < 0.05 and ^##^ *P* < 0.01 versus control; *P < 0.05 and **P < 0.01 versus model; KW: kidney weight; BW: body weight.

**Table 3:** Effects of ZWT on NF-*κ*B p65 and I*κ*B mRNA expression (*n* = 10, mean ± SD).

Groups	NF-*κ*B p65 mRNA	I*κ*B mRNA
Control	0.316 ± 0.043**	3.268 ± 0.781**
Model	1	1
ZWT (24.0 g/kg)	0.560 ± 0.154**	1.587 ± 0.131**
ZWT (12.0 g/kg)	0.736 ± 0.236*	1.271 ± 0.208*
DXM (0.9 mg/kg)	0.472 ± 0.116**	2.201 ± 0.272*

*P < 0.05 and **P < 0.01 versus model.
